# Does *Tribolium brevicornis* Cuticular Chemistry Deter Cannibalism and Predation of Pupae?

**DOI:** 10.1673/031.011.11501

**Published:** 2011-09-12

**Authors:** T Alabi, J Dean, JP Michaud, F Verheggen, G Lognay, E Haubruge

**Affiliations:** ^1^ Department of Functional and Evolutionary Entomology, Gembloux Agro-Bio Tech, University of Liege- Passage des Déportés, 2 B-5030 Gembloux, Belgium; ^2^ Penn State University, College of Agricultural Sciences, Entomology Department; ^3^ Department of Entomology, Kansas State University, Agricultural Research Center, Hays, Kansas; ^4^ Department of Analytical Chemistry, Gembloux Agro-Bio Tech, University of Liege- Passage des Déportés, 2 B-5030 Gembloux, Belgium

**Keywords:** cuticular hydrocarbons, feeding deterrence, flour disks, pupal defense

## Abstract

The cuticular hydrocarbons of insects are species-specific and often function as semiochemicals. The activity of *Tribolium brevicornis* cuticular hydrocarbons as feeding deterrents that ostensibly function to prevent pupal cannibalism and predation was evaluated. The cuticular hydrocarbons of *T. brevicornis* pupae were characterized and flour disk bioassays conducted with individual and combined extract components incorporated into artificial diets on which *Tribolium* adults fed for six days. Feeding by *T. brevicornis* and *T. castaneum* on flour disks containing cuticular extracts of *T. brevicornis* pupae resulted in reduced consumption and weight loss relative to feeding on control flour disks. In both cases, feeding deterrence indices exceeded 80% suggesting that *T. brevicornis* cuticular hydrocarbons could function to deter cannibalism and predation of pupae by larvae and adult beetles. Sixteen different cuticular hydrocarbons were identified in *T. brevicornis* pupal extracts. Eight of the commercially available linear alkanes were tested individually in feeding trials with eight *Tribolium* species. One compound (C28) significantly reduced the amount of food consumed by three species compared to control disks, whereas the compounds C25, C26, and C27 elicited increased feeding in some species. Four other compounds had no effect on consumption for any species. When four hydrocarbon mixtures were tested for synergistic deterrence on *T. brevicornis* and *T. castaneum,* none significantly influenced consumption. Our results indicate that the cuticular chemistry of *T. brevicornis* pupae could serve to deter predation by conspecific and congeneric beetles.

## Introduction

Since all life depends on the consumption of other life, almost all organisms on earth are prey for at least one other species. Predation is a mechanism of natural selection that tends to favor individuals that are more difficult to find, capture, subdue, and consume (Hoback et al. 2006). Consequently, many insects have evolved a diversity of strategies to defend against predation, including crypsis, aposematism or mimicry, structural armor, and toxic chemistry (Dettner 1987). Many defenses are mediated by the insect cuticle that is the sclerotized keratin exoskeleton that provides a barrier between the living tissue of an insect and its environment (Kolattukudy 1976; Andersen 1979). The outer layer of the insect cuticle is coated with complex mixtures of long-chain compounds such as hydrocarbons, waxy esters, sterol esters, ketones, alcohols, and quinones (Blomquist and Jackson 1979; Sokoloff 1974). In addition to basic functions such as preventing water loss, cuticular compounds also function to protect against attacks by microorganisms, parasites, and predators (Buckner 1993). For example, among the Coccinellidae, surface chemicals have been strongly implicated in the deterrence of egg predation by both adults and larvae of other species (Hemptinne et al. 2000; Cottrell 2004; Omkar and Gupta 2004).

The chemistry of adult flour beetles (*Tribolium spp*.) has been well-studied and is known to include compounds such as quinones, benzoquinones, hydrocarbons, ketones, and esters that have putative deterrent activity against predators, bacteria, and fungi (Roth 1941; Roth and Stay 1958; Howard and Mueller 1987; Arnaud et al. 2000; Yezerski et al. 2004). However, less is known about the possible chemical defenses in other *Tribolium* life stages. There is extensive evidence that sessile stages are frequently consumed by motile stages, such that both cannibalism and interspecific predation act as strong selective forces on *Tribolium* populations (Park 1948; Park et al. 1965; Stevens 1989; Via 1999; Alabi et al. 2008, 2009). Given the inability of pupae to escape attacks once encountered by a putative predator, selection for chemical defenses should be especially strong in this life stage compared to adults. However, in a comparative study of seven *Tribolium* species, *T. brevicornis* was the only one to exhibit signs of pupal defense, suggesting that pupal predation has been a significant force in its evolutionary history (Alabi et al. 2008). Both larvae and adults of *Tribolium* species avoided consumption of *T. brevicornis* pupae, although they readily consumed those of other species.

Although the cuticular compounds of several *Tribolium* species have been characterized (Tschinkel 1975; Lockey 1978; Hebanowska et al. 1990), little is known about their putative defensive functions. The objective of this study was to investigate the role of cuticular hydrocarbons in *T. brevicornis* pupal defense against conspecifics and other *Tribolium* species. The surface chemistry of *T. brevicornis* pupae was characterized, and cuticular extracts and individual and combined cuticular hydrocarbon components were incorporated into flour disks in order to determine their effects on the feeding behavior of both conspecific and congeneric adults.

## Materials and Methods

### Insects

All eight of the *Tribolium* species in this study (*T. anaphe, T. audax, T. brevicornis, T.* *castaneum, T. confusum, T. destructor, T. freemani*, and *T. madens*) were reared for at least six years in our laboratory under identical conditions before the experiments. The exact sources of these cultures are reported in Arnaud et al. (2005). Cultures were reared in 14.0 cm diameter Petri dishes under standardized conditions (25° C, 65% RH, dark incubators). Diet consisted of a standard medium of 50 g single-sifted fresh whole-wheat flour + 10% powdered brewer's yeast by dry weight. Adults and pupae were collected by sifting the flour.

### Solvent extraction of *T. brevicornis* pupae and GC-MS analysis

Cuticular hydrocarbons were extracted from three-day-old pupae of *T. brevicornis* by placing 50 pupae (25 males, 25 females) for 5 minutes into a vial containing 1 ml of *n*hexane and 1 ml of *n*-eicosene at 21.15 µg/ml as an internal standard (Hemptinne et al. 2000). The solvent extract was filtered through glass wool and transferred to another vial. Extracts were dried under nitrogen and then dissolved in 500 µl of *n*-hexane. Five groups of 50 pupae were extracted in this manner.

Compounds in the extracts were characterized with gas chromatography on a HewlettPackard HP5890 (www.hp.com) equipped with a HP-5 cross-linked 5% phenylmethylpolysiloxane capillary column (30 m × 0.25 mm, 0.25 µm film thickness) and flame ionization detector (FID). Samples were injected with a splitless injector at 280° C and carried through the column via helium gas (1.3 ml/minute). The oven temperature increased from 40 to 250° C at a rate of 8° C/minute and then to 280° C at 15° C/minute, with a final hold of 280° C for 15 minutes. Quantification was made by comparison of the peak areas with those of the internal standards. Compounds were identified with a Hewlett-Packard HP5973 mass spectrometer coupled to a HP5890 series II gas Chromatograph by comparing retention times with those of known standards and confirmed by fragmentation patterns using an electron impact ionization of 70 eV.

Compounds were assigned numbers based on the chemical nomenclature system of the International Union of Pure and Applied Chemistry (IUPAC). IUPAC organic nomenclature has three basic parts: the substituent, carbon chain length, and chemical termination. Acyclic hydrocarbons, (un) saturated, or (un)branched chain compounds are represented first by the total number of carbon atoms. Thus, C30 (Triacontane) represents an alkane with thirty carbon atoms. Mixtures of hydrocarbons were designated with the letter M and the numbered in sequence (Mixture one = M1).

### Flour disk preparation

To test the deterrence of *T. brevicornis* pupal cuticular hydrocarbons, we added hexane extracts and chemical standards to flour disks prepared as in Xie et al (1996). For the first bioassy that examined the activity of cuticular washes, 20 µl of the hexane extract was added to 80 µl of the wheat flour solution at 0.25 g/ml. The mixture was poured into new 9.0 cm diameter polystyrene Petri dishes and left to dry overnight at room temperature. Bioassays 2 and 3 utilized synthetic hydrocarbons of ∼ 99% purity (Sigma Aldrich, www.sigmaaldrich.com) in concentrations corresponding to those found in *T. brevicornis* pupal extracts (Table 1). Although the cuticular hydrocarbon extract contained both linear and methyl-branched saturated hydrocarbons (Table 1), only linear forms were available from the manufacturer for use in the experiments. Control flour disks were prepared with similar amounts of *n*hexane, wheat flour, and water as the treated disks. Once prepared, the disks were maintained at 25° C and 65% RH for 24 hours to allow *n*-hexane evaporation and to stabilize moisture content.

**Table 1.**
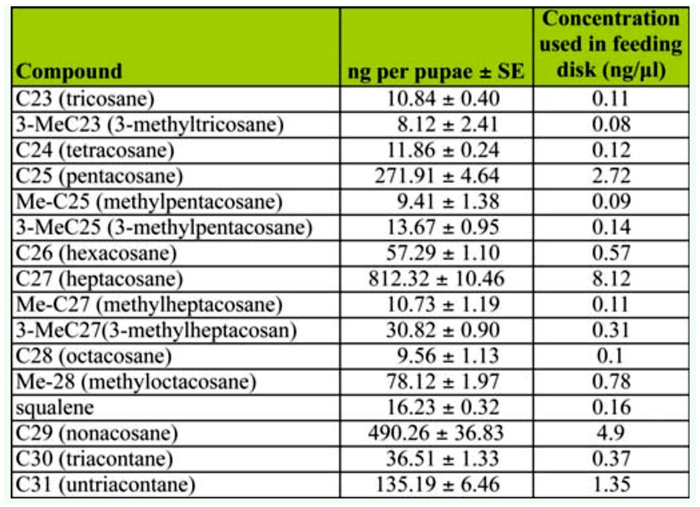
Mean quantities of cuticular hydrocarbons extracted from pupae of *Tribolium brevicornis* and concentrations used in preparation of feeding disks.

### *Tribolium* bioassays

**Figure 1.**
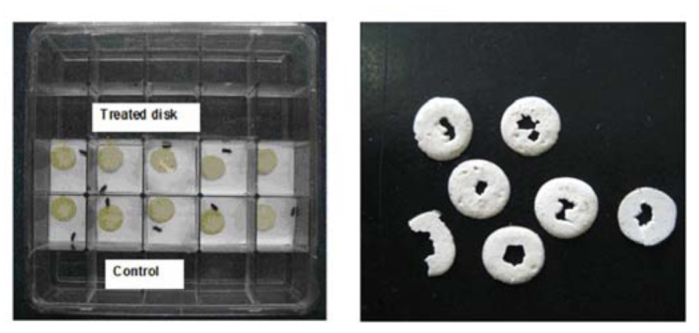
(A) Layout of feeding trials with adult beetles (*Tribolium castaneum* shown) and (B) examples of flour disks after six days of feeding. Trials were conducted in a darkened incubator set to 25° C and 65% RH. High quality figures are available online.

All bioassays were conducted with adult beetles of ∼ 30 days and with equal numbers of males and females in five replications per treatment. Beetles were starved for 48 hours prior to testing and weighed to the nearest 10-^4^ mg using a Sartorius Supermicro Balance (Sartorius Instruments, www.sartorius.com) before being transferred to 4 cm^2^ individual culture plate cells (Figure 1) (Sigma Aldrich) containing flour disks of known weights. Treatment and control trials were conducted simultaneously in separate culture plates. After six days, insects and flour disks were weighed again to determine consumption and changes in insect weights.

The first bioassay tested the activity of cuticular extracts from *T. brevicornis* pupae on diet consumption by two species: conspecifics and *T. castaneum.* The second tested the individual activity of eight linear hydrocarbons identified from the cuticular extract on eight species of *Tribolium* (*T. anaphe, T. audax, T. brevicornis, T. castaneum, T. confusum, T. destructor, T. freemani*, and *T. madens*), resulting in 128 treatments: 8 species × 8 compounds × 2 disk treatments, treatment, and control. The third bioassay was conducted to test for synergistic activity of individual cuticular hydrocarbons on *T. brevicornis* and *T. castaneum.* Due to the large number of possible combinations, we used Minitab software (2007) to generate a fractional factorial design with eight factors, corresponding to the eight hydrocarbons tested, 32 runs, and a fraction level of . Various combinations of chemicals were suggested by the software. From this design, four mixtures of compounds were selected as treatments: Ml = C23 + C24; M2 = C24 + C25 + C26; M3 = C24 + C25 + C26 + C27; M4 = C26 + C27 + C29 + C31).

The methods described by Waldbauer (1968) and Wang (1997) were used to calculate nutritional indices following each bioassay. Waldbauer's formula, described below, allows for the determination of the change in body mass (CBM) and the feeding deterrent index (FDI) of insects according to the chemicals used in these experiments,
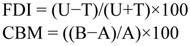

where (A) is the dry weight of the insect before the experiment, (B) is the dry weight of the insect after the experiment, (U) is the weight of the control flour disk consumed, and (T) is the weight of the treated flour disk consumed.

### Data analysis

Data derived from the experiments were logarithmically transformed (log_10_ = ln(a)/ln(10)), where a = food consumption. For each species, a one-way ANOVA was performed to compare mean food consumption among treatment and control samples. Due to inherent differences among species in body size, variation in consumption among species was analyzed by ANCOVA with food consumption as the independent variable and body size as the covariate.

## Results

### Cuticular hydrocarbons from *T. brevicornis* pupae

The hydrocarbon mixtures extracted from *T. brevicornis* pupae with hexane were composed of linear and methyl-branched saturated alkanes and squalene (Table 1). Although 16 different compounds were identified by GC-MS, three linear alkanes (C25, C27, and C29) constituted 79% of the total hydrocarbon composition.

### Activity of cuticular extracts from *T. brevicornis* pupae on adult conspecifics and *T. castaneum*

**Figure 2.**
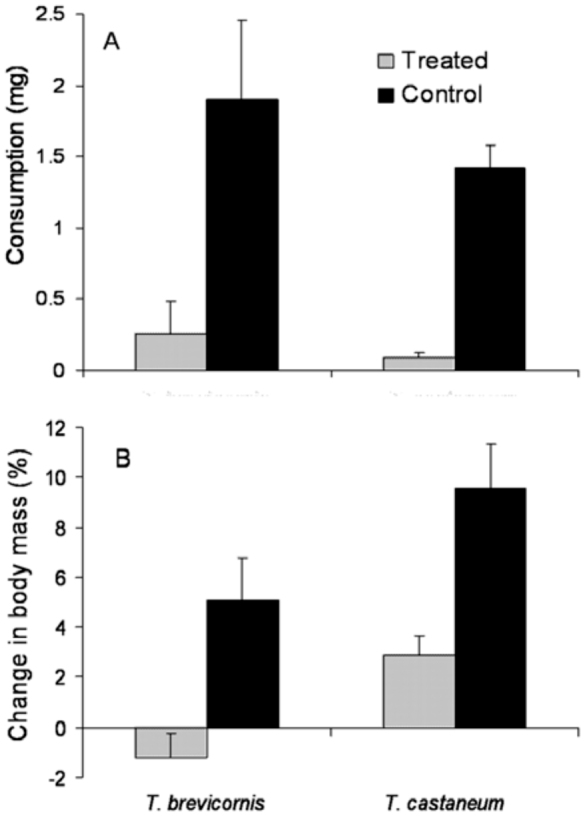
Mean (+SEM) consumption (A) and change in body mass (B) by adults of two *Tribolium* species that fed for six days on flour disks treated with hexane extracts of *T. brevicornis* pupae (shaded columns) or control disks (solid columns). High quality figures are available online.

Cuticular hydrocarbons washed from *T. brevicornis* pupae reduced flour disk consumption by both conspecifics and *T. castaneum*, producing feeding deterrent indices greater than 80% for each (*T. brevicornis*: 80.2% ± 3.7; *T. castaneum:* 88.1% ± 15.8). Within each species, beetles fed on control flour disks significantly more than on treated flour disks (Figure 2a; *T. brevicornis*: *F*_1,8_ = 7.42, *p* < 0.05; *T. castaneum*: *F*_1,8_ = 63.69, *p* < 0.01). Beetles that were fed cuticular hydrocarbon-treated flour disks also lost body weight during the course of the experiment (Figure 2b; *T. brevicornis*: *F*_1,8_ = 7.42, *p* < 0.05; *T. castaneum*: *F*_1,8_ = 12.68, *p* < 0.01).

No significant differences were found among species in consumption of either treatment or control flour disks when consumption was adjusted for differences in body weight (treatment: ANCOVA, *F*_1,7_ = 1.93, *p* = 0.20; control: ANCOVA, *F*_1,7_ = 4.60, *p* = 0.07). However, when beetles were fed treated flour disks, the CBM of *T. castaneum* was significantly higher than that of *T. brevicornis* (ANCOVA, *F*_1,7_ = 9.48, *p* < 0.05). In contrast, there was no significant difference between the CBM of *T. castaneum* and *T. brevicornis* on untreated disks (ANCOVA, *F*_1,7_ = 0.15, *p* = 0.71).

### Effects of individual synthetic hydrocarbons on feeding by several *Tribolium* species

**Figure 3.**
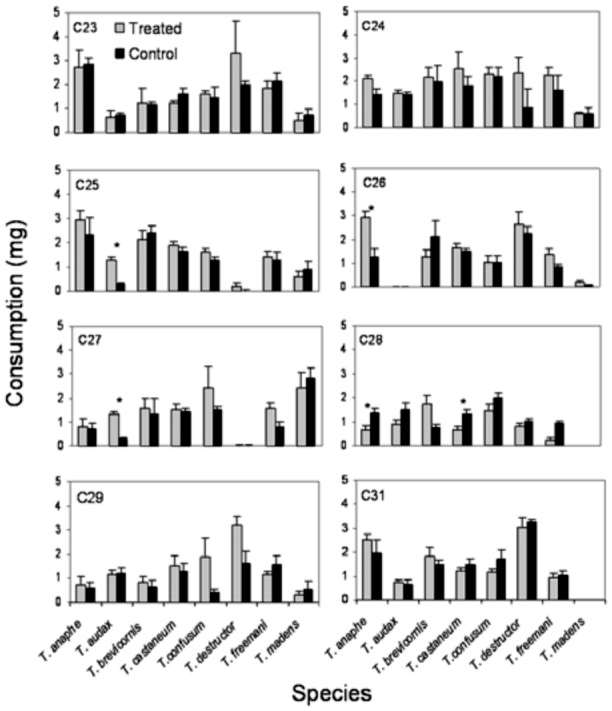
Mean (+SEM) weight of food consumed by adults of eight *Tribolium* species that fed for six days on flour disks treated with various synthetic linear hydrocarbons identified in *T. brevicornis* pupal extracts (shaded columns) or untreated controls (solid columns). Asterisks indicate significant differences (ANOVA, a = 0.05). Data missing for *T. audax* — C26 and *T. madens* — C28 and C31. High quality figures are available online.

There was substantial variation among species in consumption of flour disks treated with individual cuticular hydrocarbon components of *T. brevicornis* pupal extracts. When adjusted for body weight, interspecific comparisons revealed differences in consumption among species for each compound tested (Figure 3; ANCOVA, *p* < 0.05 for all compounds). One compound, C28, significantly reduced consumption by three species in comparison to control disks (Figure 3; *T. audax*: *F*_(1,8)_ = 7.20, *p* < 0.05; *T. castaneum*: *F*_(1,8)_ = 7.65, *p* < 0.05; *T. freemani*: *F*_(1,8)_ = 30.10, *p* < 0.01). Three other compounds elicited increased feeding in comparison to control disks in certain species: C25 (*T. audax*: *F*_(1,8)_ = 32.31, *p* < 0.01), C26 (*T. anaphe*: *F*_(1,8)_ = 9.68, *p* < 0.05), and C27 (*T. audax*: *F*_(1,8)_ = 30.31, *p* < 0.01; *T. freemani*: *F*_(1,8)_ = 6.55, *P* < 0.05). The remaining compounds did not significantly affect consumption by any species. Overall, feeding deterrence indices were less than 56% for each species per compound tested, suggesting low deterrence activities of individual compounds (Table 2).

**Table 2.**
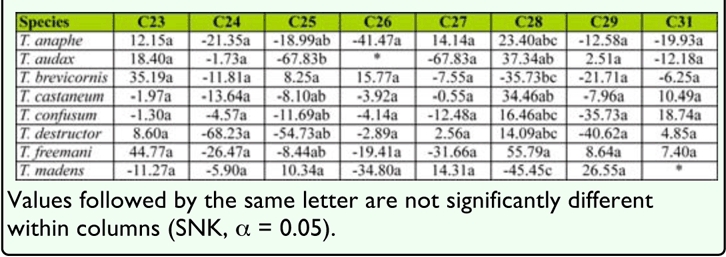
Feeding deterrence indices of flour disks impregnated with various synthetic hydrocarbons for adults of eight *Tribolium* species.

### Effects of synthetic hydrocarbon mixtures on feeding by *T. brevicornis* and *T. castaneum*

**Figure 4.**
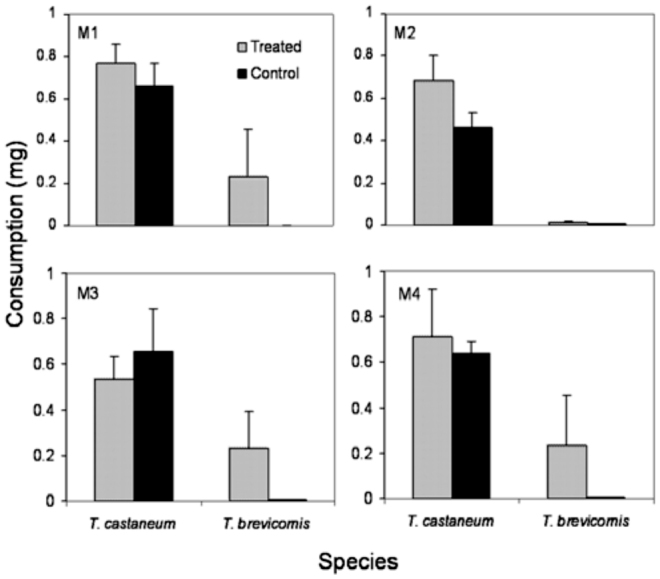
Mean (+SEM) weight of food consumed by adults of two *Tribolium* species that fed for six days on flour disks treated with mixtures of synthetic linear hydrocarbons (shaded columns) identified in T. *brevicornis* pupal extracts (M1 = C23 + C24; M2 = C24 + C25 + C26; M3 = C24 + C25 + C26 + C27; M4 = C26 + C27 + C29 + C31) and untreated controls (solid columns). High quality figures are available online.

Since chemical mixtures can be more biologically active than isolated compounds, we tested a subset of possible combinations of hydrocarbons identified from *T. brevicornis* pupae on the feeding of conspecifics and *T. castaneum.* None of the four hydrocarbon mixtures significantly influenced consumption differences between treated and control flour disks for either species (Figure 4; *p* > 0.05 in all cases). All insects lost weight during the experiment (Table 3). Additionally, there were no differences in weight lost between *T. castaneum* and *T. brevicornis* for any mixture (ANCOVA; M1: *F*_1,9_ = 0.12, *p* = 0.74; M2: *F*_1,9_ = 0.25, *p* = 0.63; M3: *F*_1,9_ = 0.13, *p* = 0.73; M4: *F*_1,9_ = 0.15, *p* = 0.71). Deterrence rates were less than 11% and mostly negative, suggesting no deterrent activity (Table 3).

## Discussion

Cuticular surface chemicals are known to convey information in many inter- and intraspecific interactions including species and nestmate recognition, assessment of mate suitability, dominance status, and overall individual fitness (Howard and Blomquist 2005). When hexane extracts of *T. brevicornis* pupae were added to flours disks, consumption by conspecific and *T. castaneum* adults were significantly reduced compared to controls. Thus, *T. brevicornis* cuticular hydrocarbons appear to deter feeding by both conspecific and congeneric adults, these results consistent with the inference of their functionality in pupal defense against cannibalism and predation as previously demonstrated by Alabi et al. (2008). Adult *T. brevicornis* consumed less of the treated flour disks per gram of body weight than did adult *T. castaneum,* suggesting that the cuticular extract had a stronger deterrent effect on conspecifics than on other species. This is in contrast to the deterrent effect of cuticular hydrocarbons on the eggs of Coccinellidae that tend to have greater heterospecific than conspecific activity (Hemptinne et al. 2000; Cottrell 2004). Therefore, it is possible that cannibalism has been a stronger selective force than interspecific predation in the evolution and expression of cuticular hydrocarbon feeding deterrents in *T. brevicornis* pupae.

**Table 3.**
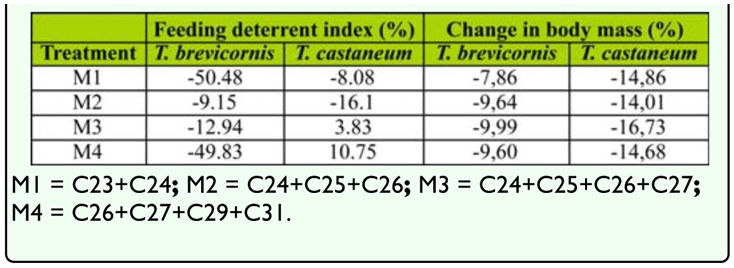
Feeding deterrence indices of flour disks impregnated with four mixtures of linear hydrocarbons for adults of two *Tribolium* species and changes in body mass relative to adults fed untreated control disks.

The individual components of *T. brevicornis* cuticular extract that we tested varied widely in their effects on different *Tribolium* species. Most of the 64 compound × species combinations showed no difference in consumption of treated and control flour disks, though some trials revealed feeding inhibition. Although C28 was present in low concentration in the cuticular extract of *T. brevicornis* pupae, it was the only chemical that reduced consumption by three *Tribolium* species. Other compounds (C25, C26, and C27) actually stimulated feeding by some species compared to controls. The activity of semiochemicals in bioassays can also be strongly dependent on the concentration tested. For example, Verheggen et al. (2007) demonstrated concentration-dependent activity of benzoquinones from *T. confusum*; low concentrations were attractive to adults of both sexes, higher concentrations were deterrent. Thus, it is conceivable that different results could be obtained with different concentrations of the same compounds.

While many laboratory experiments have examined the effects of semiochemicals in isolation, there is mounting evidence that biological activity can depend on the additive or synergistic activities of combinations (Hay et al. 1994; Nelson and Kursay 1999; Dyer et al. 2003). For example, the queen mandibular pheromone of the honeybee, *Apis mellifera,* comprises five compounds that are active only when they occur together (Keeling et al. 2003). Only a small subset of the possible combinations of compounds present in cuticular extracts was tested for effects on *T. brevicornis* and *T. castaneum* feeding behavior, and it is possible that other combinations might demonstrate additive or synergistic activity. Similarly, other compounds in the extract, such as methyl branched hydrocarbons were not commercially available and could be essential components of an active mixture.

Although intrinsically vulnerable, the pupae of many holometabolous insects have evolved defensive mechanisms that may be behavioural, physical, or chemical in nature. For example, pupae of some tenebrionid species rotate their abdominal segments upon tactile stimulation to effectively deter predation and cannibalism (Ichikawa and Kurauchi 2009). Pupae of certain coccinellid species possess “gin traps” that can be employed to sever the antennae of potential predators such as ants (Eisner and Eisner 1992). Pupae of the small cabbage white, *Pieris rapae*, retain alkaloid-secreting glandular hairs that develop during the larval stage (Smedley et al. 2002). Chemical defenses can be costly to produce, often requiring a trade-off with respect to other defensive strategies. Notably, pupae of *T. brevicornis* require nine days to complete development in comparison to six days for *T. castaneum* (Arnaud et al. 2005). Although many factors may be responsible for speciesspecific developmental times, longer development means extended exposure to predation risk. Two divergent developmental strategies may prevail in *Tribolium* spp.: (i) rapid pupal development with minimal investment in defense and (ii) slower development with greater investment in defense.

In summary, this study provides evidence that cuticular hydrocarbons expressed by *T. brevicornis* pupae have a feeding deterrent effect on both conspecific and congeneric adults and thus may serve to defend pupae against both cannibalism and predation. Bioassays with individual components of the extract and subsequent selected combinations generated mixed results that in no case approached the level of deterrency (> 80%) obtained with the crude extract. This suggests that many different compounds contribute, either additively or synergistically, to the defensive chemistry of pupae.
